# Comparison of common acute respiratory infection case definitions for identification of hospitalized influenza cases at a population-based surveillance site in Egypt

**DOI:** 10.1371/journal.pone.0248563

**Published:** 2021-03-25

**Authors:** Emily Rowlinson, Lisa Peters, Adel Mansour, Hoda Mansour, Nahed Azazzy, Mayar Said, Sahar Samy, Eman Abbas, Hanaa Abu Elsood, Manal Fahim, Alaa Eid, Erik Reaves, Chris Van Beneden, Sarah Hamid, Sonja Olsen, Julia Fitzner, Erica Dueger

**Affiliations:** 1 U.S. Naval Medical Research Unit-3, Cairo, Egypt; 2 Egyptian Ministry of Health and Population, Cairo, Egypt; 3 U.S. Centers for Disease Control and Prevention, Atlanta, Georgia, United States of America; 4 World Health Organization, Geneva, Switzerland; 5 Sanofi Pasteur, Medical Evidence Generation, Lyon, France; The University of Hong Kong, CHINA

## Abstract

**Background:**

Multiple case definitions are used to identify hospitalized patients with community-acquired acute respiratory infections (ARI). We evaluated several commonly used hospitalized ARI case definitions to identify influenza cases.

**Methods:**

The study included all patients from a population-based surveillance site in Damanhour, Egypt hospitalized for a broad set of criteria consistent with community acquired ARIs. Naso- and oropharyngeal (NP/OP) swabs were tested for influenza using RT-PCR. Sensitivity, specificity and PPV for influenza identification was compared between the 2014 WHO Severe Acute Respiratory Infection (SARI) definition (fever ≥38°C and cough with onset within 10 days), the 2011 WHO SARI definition (fever ≥38°C and cough with onset within 7 days), the 2006 PAHO SARI definition, the International Emerging Infections Program (IEIP) pneumonia case definition, and the International Management of Childhood Illness (IMCI) case definitions for moderate and severe pneumonia.

**Results:**

From June 2009-December 2012, 5768 NP/OP swabs were obtained from 6113 hospitalized ARI patients; 799 (13.9%) were influenza positive. The 2014 WHO SARI case definition captured the greatest number of ARI patients, influenza positive patients and ARI deaths compared to the other case definitions examined. Sensitivity for influenza detection was highest for the 2014 WHO SARI definition with 88.6%, compared to the 2011 WHO SARI (78.2%) the 2006 PAHO SARI (15.8%) the IEIP pneumonia (61.0%) and the IMCI moderate and severe pneumonia (33.8% and 38.9%) case definitions (IMCI applies to <5 only).

**Conclusions:**

Our results support use of the 2014 WHO SARI definition for identifying influenza positive hospitalized SARI cases as it captures the highest proportion of ARI deaths and influenza positive cases. Routine use of this case definition for hospital-based surveillance will provide a solid, globally comparable foundation on which to build needed response efforts for novel pandemic viruses.

## Introduction

The World Health Organization’s (WHO) 2004 Global Burden of Disease Survey identified lower respiratory tract infections as the leading cause of infectious disease mortality worldwide, causing 4.2 million deaths annually [1]. It is estimated that over two million children under the age of five die of pneumonia yearly [2]. Recent modeling estimates indicate that seasonal influenza accounts for up to 645, 832 of these deaths every year [3]. Prior to 2009, global influenza surveillance systems focused primarily on outpatient influenza-like illness surveillance to monitor circulating influenza strains and inform vaccine strain selection. The 2009H1N1 and the ongoing SARS-CoV-2 pandemics highlight the need to support development of a robust global influenza surveillance for regular disease burden and severity estimates, as these systems can provide the foundation for systems that can be used to rapidly assess the pandemic potential of emergent novel viruses [4].

Burden of disease (BOD) estimates for influenza and other respiratory pathogens at local, national, regional, and global levels are calculated using data from hospital-based acute respiratory infection (ARI) surveillance systems. BOD estimates for several consecutive years provide baseline data that help to identify the relative scale of yearly outbreaks, potential changes in disease severity and/or alterations in high risk populations such as pregnant women or the elderly. These data ultimately influence policy maker resource recommendations for response efforts by allowing often limited quantities of vaccines and antivirals to be targeted to the most at risk populations. Producing accurate and comparative burden of disease estimates from national surveillance data relies on a case definition that captures the majority of severe disease in a given population to generate standardized, consistently representative epidemiological profiles of influenza cases.

Sentinel surveillance systems are also critical for providing data for disease severity estimates, further supporting pandemic preparedness and response. In the event of a severe epidemic and/or emergence of a novel virus, tools such as WHO’s Pandemic Influenza Severity Assessments (PISA) exist to assess the transmissibility of the virus, the severity of disease, and the impact on the healthcare system. Impact is measured by the number of people accessing care, and severity is determined by the number of hospitalized patients and transmissibility is the degree of person-to-person spread [5]. When standardized case definitions are used across sites and accurate case estimates are available, such assessments can be used for quick and appropriate response decisions.

To address pandemic risk assessments needs the WHO recommends establishing sentinel surveillance for hospitalized patients using a standardized Severe Acute Respiratory Infection (SARI) case definition (see Table 1) [6]. In 2011, the WHO proposed a new SARI case definition to increase sensitivity and facilitate implementation [7]. Based on data received from sites piloting the 2011 definition, it was further revised in 2014 to expand the number of days of symptom onset from seven to ten. This decision was in part supported by data from a population-based surveillance site in Egypt [8], reported for the first time herein; this paper will describe the results of an analysis comparing the proportion of influenza positive patients and severe disease outcomes captured (e.g. ICU admission, ventilation required and death) using several different case definitions: the 2014 WHO SARI case definition, the 2011 WHO SARI case definition, the 2006 Pan American Health Organization (PAHO) SARI definition, the International Emerging Infections Program (IEIP) pneumonia case definition, and the International Management of Childhood Illness (IMCI) case definitions for moderate and severe pneumonia in children >31 days to <5 years.

**Table 1:** Case definitions used for analysis.

Used for all hospitalized patients age >31 days				Used for hospitalized patients age >31 days—<5 years	
**2006 PAHO SARI**:	**2011 WHO SARI**:	**2014 WHO SARI**:	**CDC-IEIP**:	**IMCI Severe**:	**IMCI Moderate**:
Current fever ≥ 38°C*	Current fever ≥ 38°C* or history of fever with this illness	Current fever ≥ 38°C* or history of fever with this illness	Current Fever ≥ 38°C* or Hypothermia or Abnormal White Blood Cell Count	Tachypnea, Cough, or Difficulty Breathing	Tachypnea
AND	AND	AND	AND	AND	AND
Cough or Sore Throat	Cough	Cough	One of the Following:	One of the Following:	Cough or Difficulty Breathing
AND	Symptom onset in the last 7 days	Symptom onset in the last 10 days	• Tachypnea	• Unable to Drink/Breastfeed	
Shortness of Breath or Difficulty Breathing			• Cough	• Lethargic/Unconscious	
			• Abnormal Breath Sounds	• Vomiting	
				• Convulsions	
				• Nasal Flaring	
			• Sputum Production	• Grunting	
			• Hemoptysis	• Oxygen Saturation <90%	
				• Chest Indrawing	
			• Chest Pain	• Stridor	
AND			• Dyspnea		
Requiring hospital admission					

*History of fever was added to case definition criteria for purposes of enrollment; however, analysis was performed using case definitions as they are specified here.

## Methods

### Screening and enrollment

Population-based surveillance for Acute Respiratory Infection (ARI) was initiated at all three governmental referral hospitals in Damanhour District, Arab Republic of Egypt. Damanhour is a mixed urban/rural area in Egypt’s Nile Delta Region with a population of over half a million; further details of population and demographic characteristics of Damanhour district have been described previously [9–11]. Previously published data from this population demonstrated a high incidence of hospitalization for ARI with a defined winter seasonality and identified influenza and RSV as the largest contributors to hospitalized ARI in adults and children, respectively [9]. Screening forms and questionnaires (S1 and S2 Files) were developed in conjunction with local staff and validated prior to use in surveillance. Translation and back-translation of these forms was conducted by study staff fluent in Arabic and English and certified prior to study implementation. Hospitalized patients were eligible if they met a broad set of symptomologies indicative of severe respiratory infection [9] (S1 File). During data analysis, patients were retrospectively grouped according to whether they met each of the five case definitions described in Table 1. Patients were excluded if they were <31 days of age, not a resident of Damanhour district, or had already been enrolled in the surveillance system for the current episode of illness. Patients <31 days of age were excluded because study physicians were not comfortable performing nasopharyngeal (NP) swabs on ill infants; persons not a resident of Damanhour district were excluded because they were not a member of the underlying population for which the population-based surveillance system was designed to capture; those who had already been enrolled were excluded to prevent double-counting of the same episode. After consent was obtained from the patient or their legal guardian, study personnel conducted patient interviews and chart reviews to complete questionnaires detailing demographic variables, relevant medical history, and symptomology of the patients’ current illness (S2 File). No estimates of influenza vaccine coverage in Egypt have been published to date; however, less than 1% of the study population reported receiving influenza vaccination in the past 12 months, and no patients reported receiving pneumococcal vaccine.

### Specimen collection and testing

NP and oropharyngeal (OP) specimens were collected from enrolled hospitalized patients and placed in liquid viral transport media. Specimens were tested at a central reference laboratory in Damanhour and an aliquot of each specimen was stored in liquid nitrogen and shipped weekly to the U.S. Naval Medical Research Unit No. 3 (NAMRU-3) reference laboratory in Cairo, Egypt for confirmation. All specimens were tested for the presence of Ribonuclease P (RNP) as an indicator of sufficient human cellular material for viral pathogen testing. Specimens were tested by reverse transcription, real time PCR (rRT-PCR) assays for influenza A and B using CDC developed assays and testing protocol. Specimens positive for influenza A were further subtyped for seasonal H1N1, seasonal H3N2, H1N1pdm09 and H5N1. Although this study tested specimens for multiple pathogens including RSV [9, 10], this analysis was limited to influenza due its severity and pandemic potential.

### Data management and statistical analyses

Data collected were reviewed by clinical supervisors and senior level investigators for consistency and errors and double-entered using Microsoft Access. Statistical analyses were conducted using Stata 11 [12] and SPSS version 16.0 [13].

The number of enrolled patients, ARI deaths, and patients positive for influenza captured by each case definition were compared to that of the total enrolled study population, stratified by the following age groups: <5, 5-<65, ≥65. Sensitivity, specificity, and positive and negative predictive value for influenza detection for each of the case definitions were calculated and compared across influenza subtypes. Specificity was assessed as an indicator of the burden on laboratories; low specificity would indicate that the syndromic case definition includes many persons who do not have influenza virus infection and thus more laboratory resources dedicated to testing of persons who do not truly have influenza-virus infection. PPV/NPV are influenced by population prevalence of influenza and other pathogens that manifest as ARI and thus a useful metric for detection of emerging epidemics of other respiratory pathogens. Additionally, we compared characteristics of cases missed by the 2011 and 2014 WHO SARI case definitions.

### Ethics statement

This study was approved by Scientific and Institutional Review Boards (IRB) (protocol #906) at NAMRU-3 in Cairo, Egypt, and the IRB (protocol #5641) at the Centers for Disease Control and Prevention in Atlanta, GA, USA. The protocol also received approval from the Egyptian Ministry of Health and Population in Cairo. A waiver of documentation of consent from patients or their guardians was obtained from both IRBs. Surveillance staff verbally described all study procedures to the patients and provided them with a written document detailing all procedures; study staff then obtained verbal consent from patients over 17 years of age, verbal assent and parental consent for patients 5–17 years of age, and parental/guardian consent for patients <5 years of age. Study staff signed consent forms documenting verbal consent/assent from patients/guardians and a record of completed consent procedures was maintained for all patients. All study forms used for screening and data collection were reviewed and approved by both IRBs prior to study implementation.

## Results

**Fig 1:**
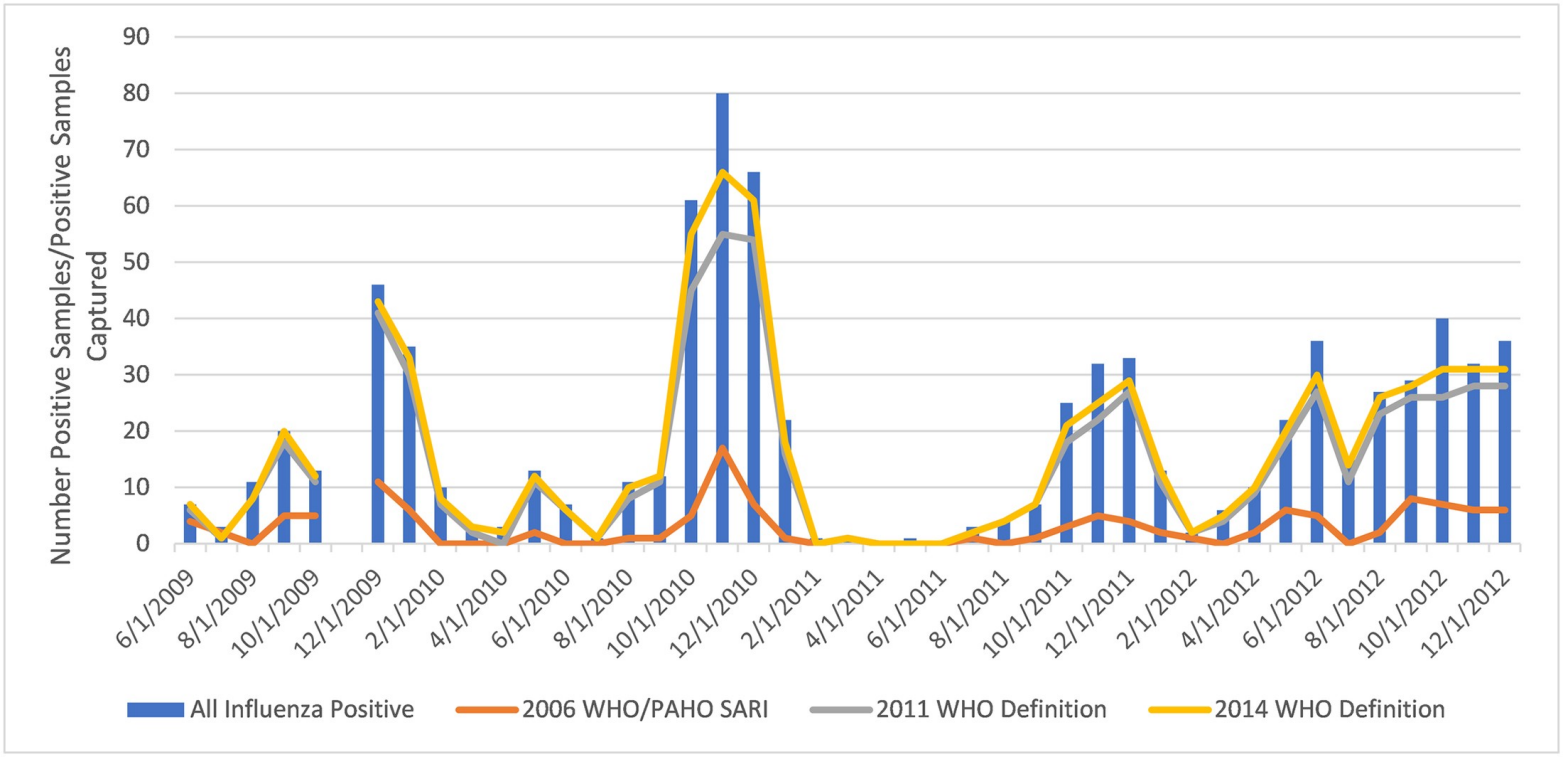
Comparison of influenza epidemiological curves for June 2009-December 2012 using the 2006 PAHO/WHO SARI, 2011 and 2014 WHO SARI case definitions.

Between June 2009 and December 2012, a total of 11,929 inpatients with possible ARI were screened for eligibility. Of those screened, 6,684 (56.0%) were eligible for the study and 6,113 (91.4%) of eligible patients consented to enrollment. The majority (54.2%) of enrolled patients were male. NP/OP swabs were obtained and tested for 5,768 (94.3%) enrolled patients, of which 799 (13.9%) were positive for influenza A or B. Three persons with a data discrepancy indicating an NP/OP swab was not collected but with an influenza-positive result were not included in the total count of influenza positives. The 2014 WHO SARI case definition captured as many or more influenza positive cases than the 2011 WHO SARI case definition and the 2006 WHO/PAHO SARI case definition across all years of surveillance (Fig 1).

Of the six case definitions compared in this study, the 2014 WHO SARI captured the greatest percentage of the total enrolled ARI cases for all three age groups with 88.1% for <5, 82.6% for 5-<65, and 77.6% for ≥65 (Table 2). Overall, the 2014 SARI case definition also captured 10.4% more influenza-positive patients and 4% more deaths than the 2011 SARI case definition. Sensitivity for influenza detection was highest for the 2014 WHO SARI definition with 88.6%, compared to the 2011 WHO SARI (78.2%) the 2006 PAHO SARI (15.8%) the IEIP pneumonia (61.0%) and the IMCI moderate and severe pneumonia (33.8% and 38.9%) case definitions (IMCI applies to <5 only). There were 50 (0.8%) deaths among enrolled patients with ARI, 8 (16%) of which occurred in children <5 years, 18 (36%) in those 5-<65 and 24 (48%) in those > = 65; none of the case definitions captured all ARI deaths (Table 2). Of 42 deaths in patients ≥5 years, 3 were influenza A positive (2 H1N1 and 1 H3N2) and none were influenza B positive; among 8 deaths in patients <5 years, none were influenza positive. No single case definition captured all three influenza-positive deaths.

**Table 2:** Acute Respiratory Illness (ARI) patients captured by case definitions.

	IMCI Moderate	IMCI Severe	CDC-IEIP	2006 WHO/PAHO SARI	2011 WHO SARI (≤7 days symptom duration)	2014 WHO SARI (≤10 days symptom duration)
Enrolled ARI Patients Captured (%, 95% C.I.)*						
Total (N = 6113)	-	-	49.8% (48.6–51.01)	17.0% (16.1–17.9)	73.2% (71.2–73.4)	84.2% (83.3–85.1)
<5 years (N = 2193)	55.1% (53.0–57.2)	57.9% (55.8–60.0)	61.1% (59.1–63.2)	29.4% (27.4–31.3)	80.8% (79.2–82.4)	88.1% (86.7–89.4)
5-<65 years (N = 3283)	-	-	45.0% (43.3–46.7)	9.6% (8.6–10.6)	69.3% (67.8–70.9)	82.6% (81.3–83.9)
≥65 years (N = 446)	-	-	31.2% (26.9–35.4)	13.9% (10.8–17.3)	66.1% (61.7–70.4)	77.6% (73.8–81.4)
Influenza Positive Captured (%, 95% C.I.)*						
Total (N = 799)	-	-	61.0% (57.6–64.3)	15.8% (13.3–18.3)	78.2% (75.3–81.0)	88.6% (86.4–90.7)
<5 years (N = 198)	33.8% (27.3–40.4)	38.9% (32.3–46.0)	69.7% (63.1–75.8)	26.8% (20.7–32.8)	68.7% (62.1–75.3)	80.8% (75.3–86.4)
5-<65 years (N = 555)	-	-	59.3% (55.1–63.4)	11.5% (8.8–14.2)	81.6% (78.4–84.7)	91.9% (89.5–94.1)
≥65 years (N = 46)	-	-	43.5% (30.4–58.7)	19.6% (8.7–32.6)	78.3% (65.2–89.1)	82.6% (71.7–93.5)
ICU Admissions Captured (%, 95% C.I.)*						
Total (N = 247)	-	-	57.9% (51.8–64.0)	22.3% (17.4–27.5)	74.1% (68.4–79.4)	81.8% (76.9–86.6)
<5 years (N = 100)	38.0% (29.0–48.0)	50.0% (40.0–60.0)	62.0% (52.0–71.0)	28.0% (19.0–37.0)	86.0% (79.0–92.0)	90.0% (84.0–95.0)
5-<65 years (N = 113)	-	-	53.1% (44.2–61.9)	16.8% (10.6–23.9)	70.8% (61.9–78.8)	84.1% (77.0–90.3)
≥65 years (N = 34)	-	-	58.8% (41.2–73.5)	23.5% (8.8–38.2)	50.0% (32.4–67.6)	50.0% (32.4–67.6)
ARI Deaths Captured (%, 95% C.I.)*						
Total (N = 50)	-	-	58.0% (44.0–72.0)	28.0% (16.0–40.0)	58.0% (44.0–72.0)	62.0% (48.0–76.0)
<5 years (N = 8)	87.5% (62.5–100)	75.0% (37.5–100)	37.5% (12.5–97.5)	25.0% (0–62.5)	75.0% (37.5–100)	75.0% (37.5–100)
5-<65 years (N = 18)	-	-	61.1% (38.9–83.3)	33.3% (11.1–55.6)	61.1% (38.9–83.3)	72.2% (50.0–88.9)
≥65 years (N = 24)	-	-	62.5% (41.7–79.2)	25.0% (8.3–41.7)	50.0% (29.2–70.8)	50.0% (29.2–70.8)

*C.I. = Confidence Interval.

The 2014 case definition had the highest sensitivity for influenza in all age groups (<5, 5-<65,≥65) with 80.8%, 91.9% and 82.6% respectively (Table 3); however, it also had the lowest specificity of all case definitions examined (15.6%). PPV and NPV were very similar between the 2011 and 2014 definitions with 14.7% and 88.6% for 2011 respectively and 14.4% and 89.5% for 2014.

**Table 3:** Sensitivity, specificity, Positive Predictive Values (PPV), and Negative Predictive Values (NPV) for laboratory-confirmed influenza infection by case definition.

	IMCI Moderate	IMCI Severe	CDC-IEIP	2006 WHO/PAHO SARI	2011 WHO SARI (≤7 days symptom duration)	2014 WHO SARI (≤10 days symptom duration)
Sensitivity (Estimate, 95% C.I.)*						
Total	-	-	61.0% (57.5–64.4)	15.8% (13.3–18.4)	78.2% (75.1–81.0)	88.6% (85.2–87.0)
<5 years	33.8% (27.3–40.9)	38.9% (32.1–46.1)	69.7% (62.8–76.0)	26.8% (20.7–33.5)	68.7% (61.7–75.1)	80.8% (74.6–86.0)
5-<65 years	-	-	59.3% (55.1–63.4)	11.5% (9.0–14.5)	81.6% (78.1–84.8)	91.9% (89.3–94.0)
≥65 years	-	-	43.5% (28.9–58.9)	19.6% (9.4–33.9)	78.3% (63.6–89.1)	82.6% (68.6–92.2)
Specificity (Estimate, 95% C.I.)*						
Total	-	-	51.8% (50.2–53.2)	83.1% (82.0–84.1)	27.7% (25.9–28.4)	15.6% (14.6–16.7)
<5 years	43.6% (41.3–45.9)	39.9% (37.7–42.2)	39.9% (37.6–42.1)	70.7% (68.6–72.8)	18.0% (16.3–19.8)	10.9% (9.6–12.4)
5-<65 years	-	N/A	57.7% (55.8–59.5)	91.1% (90.0–92.2)	32.5% (30.7–34.3)	18.0% (16.6–19.5)
≥65 years	-	-	69.8% (64.8–74.4)	86.9% (85.6–92.6)	34.6% (29.7–39.7)	21.8% (17.7–26.4)
Positive Predictive Value (PPV) (Estimate, 95% C.I.)*						
Total	-	-	16.9% (15.6–18.3)	13.1% (11.0–15.3)	14.7% (13.7–15.8)	14.4% (13.5–15.5)
<5 years	6.0% (4.6–7.5)	6.4% (5.1–7.9)	10.9% (9.2–12.8)	8.8% (6.7–11.4)	8.1% (6.9–9.5)	8.7% (7.5–10.1)
5-<65 years	-	-	22.2% (20.1–24.4)	20.9% (16.5–25.9)	19.7% (18.1–21.4)	18.6% (16.6–19.5)
≥65 years	-	-	15.3% (9.6–22.6)	15.8% (7.5–27.9)	13.0% (9.3–17.6)	11.7% (8.4–15.7)
Negative Predictive Value (NPV) (Estimate, 95% C.I.)*						
Total	-	N/A	89.2% (88.0–90.3)	86.0% (85.0–87.0)	88.6% (86.9–90.1)	89.5% (87.3–91.5)
<5 years	86.2% (83.8–88.3)	86.1% (83.6–88.3)	86.2% (78.2–95.3)	90.1% (88.5–91.6)	84.5% (80.5–89.8)	84.4% (79.2–88.7)
5-<65 years	-	-	87.4% (85.8)	83.5% (82.1–84.8)	89.7% (87.6–91.5)	91.6% (88.9–93.8)
≥65 years	-	-	90.8% (86.8–93.9)	89.6% (86.0–92.6)	92.7% (87.0–96.4)	90.9% (82.9–96.0)

*C.I. = Confidence Interval.

Cases missed by the 2011 and 2014 WHO case definitions are presented in Table 4. Of the 799 influenza positive cases, 92 (11.5%) were missed by the 2014 SARI definition; 81 (88%) of the 92 missed cases had symptom duration greater than 10 days, 11 (12%) had no cough, and 2 (2%) had no current or historical fever. Nine (10%) of the influenza cases missed by the 2014 case definition were receiving care in the ICU, and 3 of these presented without cough. Of the three influenza-associated deaths, neither had respiratory virus co-infections detected, one had symptom duration greater than 10 days and one had no cough. Both deaths occurred in persons over 45 years of age (46 and 75), one was female and one was male, neither had co-morbid diabetes, liver disease, or asthma. Both cases were ventilated within the ICU prior to death.

**Table 4:** Cases missed by the 2011 and 2014 WHO SARI case definitions.

	Enrolled ARI		Influenza Positive		ARI deaths		Influenza deaths	
	N = 6113		N = 799		N = 50		N = 2	
	2011	2014	2011	2014	2011	2014	2011	2014
Total cases missed	1640	966	175	92	21	19	2	2
% cases missed	26.8%	15.8%	21.9%	11.5%	42.0%	38.0%	100%	100%
Cases missed due to:								
Duration*	1,466 (89.3%)	763 (78.9%)	165 (94.3)	81 (88.0%)	5 (23.0%)	5 (26.3%)	1 (50%)	1 (50%)
No current or historical fever	49 (3.0%)	49 (5.1%)	3 (1.7%)	3 (3.3%)	6 (71.4%)	6 (31.6%)	0	0
No cough	170 (10.4%)	170 (17.6%)	11 (6.3%)	11 (12%)	9 (42.9%)	9 (47.4%)	0	0
No current or historical fever OR cough	4 (<1%)	4 (<1%)	2 (1.0%)	2 (2%)	1 (4.7%)	1 (5.3%)	0	0

* >7 days for 2011 and >10 days for 2014.

## Discussion

Data used for this comparative study were derived from a large, multi-year population-based surveillance site with the objective of capturing the majority of hospitalized ARI cases in the catchment area. One of the original objectives of the surveillance system was to evaluate the multiple case definitions used for screening hospitalized ARI patients and thus the criteria for these case definitions were included in the original surveillance design. This resulted in a large, symptomatically diverse patient database from which to calculate the proportion of cases and deaths captured and missed by each case definition along with measures of sensitivity specificity, positive and negative predictive values for capture of laboratory-confirmed influenza cases.

Of all case definitions evaluated, the 2014 WHO SARI case definition was the most inclusive for cases of severe acute respiratory infections, capturing the highest proportion of enrolled ARI patients, influenza positive cases, and ARI deaths. All other case definitions evaluated excluded a sizable proportion of influenza positive patients, especially in patients <5 years. When the 2011 case definition was expanded to include patients reporting a symptom duration of ≤10 days, the 2014 WHO SARI case definition increased sensitivity for identification of influenza positive patients by 12.1% in patients <5 years, 10.3% in patients 5-<65, and 4.3% in patients ≥65 years. This is consistent with data demonstrating that young children shed virus for a longer duration than adults [14]. The large gains in influenza sensitivity corresponded with only a marginal reduction in PPV for patients ≥5 years of age, and actually increased PPV in children <5 years (Table 3).

Several factors could explain the increase in influenza sensitivity with an increase of illness duration. Unpublished data from a healthcare utilization survey in the same population demonstrates that over half of patients who reported hospitalization for an acute respiratory infection first sought care at one or more outpatient facilities; such individuals would report a longer duration of symptoms by the time they present for hospitalization. Alternatively, these patients may have experienced chronic respiratory symptoms due to asthma or chronic obstructive pulmonary disease and later developed a secondary infection with influenza. To capture the true duration of symptoms due to an acute respiratory infection and account for similar settings where patients wait longer to seek care, it may be necessary to specify the date of onset of fever independently of cough, although this would likely be subject to recall bias.

Despite increasing the overall capture of ARI deaths by 4.0% as compared to alternative case definitions, the 2014 WHO SARI definition still excluded 19 of 50 deaths captured during surveillance, including two of the three influenza-associated deaths. Presentation without cough was an excluding factor in 6% of influenza positive cases and 43% of ARI deaths (Table 3) including both missed influenza-positive deaths. Including patients in ARI surveillance that are admitted to the ICU with evidence of respiratory infection from symptoms and clinical findings other than cough, such as abnormal breath sounds, dyspnea, or tachypnea may increase the capture of influenza infections with severe outcomes.

The 2014 WHO SARI definition confers several advantages to hospitalized ARI surveillance systems. The sensitivity of this broader case definition was higher than the other four case definitions examined, consistent with multiple studies demonstrating that the inclusion of respiratory symptoms beyond cough and history of fever does not improve sensitivity for influenza infection [15–18]. Additionally, extending the time from symptom onset to presentation increases sensitivity for a wide range of emerging respiratory pathogens, including COVID-19 [19]. This simplified case definition is also easier to implement for sentinel surveillance as the elimination of respiratory symptoms requiring a clinical assessment allows trained health workers to screen patients. Including a standardized, simplified case definition as a component of ongoing surveillance systems across multiple regions will allow for streamlined data reporting and accurate comparison of the severity of novel viruses and the potential threat of a future pandemics.

There are some challenges to implementing the 2014 WHO SARI case definition for surveillance. Research has suggested that a case definition of cough and fever may have a lower positive predictive value for influenza in children <5 years, who typically make up a large percentage of SARI cases [20]. This is often due to the fact that children may be infected by other pathogens with similar symptomology [21]. Our data support this, as the PPV of the 2014 WHO SARI case definition for children <5 years in our study was lower than in patients ≥5 years (8.7% vs. 18.6% for 5-<65 and 11.7% for ≥65), even during influenza season. The PPV for this age group is comparable to published data from Kenya (7.0%), which also demonstrated a higher PPV for cough and any fever (subjective or measured) in patients ≥5 years [22].

Utilization of a case definition with high sensitivity is essential to establishing the burden of influenza in countries and regions with limited resources and capturing severe cases that may be sentinel events for emerging infections of pandemic potential. Restricting enrollment by seasonality or use of a highly specific case definition may miss important unusual epidemiological and/or seasonal occurrences. However, capturing a larger number of cases through use of a more sensitive case definition, as suggested, may result in a significant increase in the number of samples collected, posing a greater burden on surveillance and laboratory staff. To address this, standardized sampling schemes can help to match the laboratory and/or budgetary capacity of each member state, thus allowing for the most representative sample possible with the lowest degree of bias. Utilization of such a sampling scheme prior to a pandemic will help ensure reporting is comparable and allow for quick and accurate assessments of emerging influenza subtypes and other respiratory viruses.

Our study has several limitations. The date of onset of symptoms was not recorded for a small percentage (1.0%), of ARI patients; however, none of the patients with records missing symptom duration were influenza positive or died. The date of symptom onset was likely inaccurate in a proportion of cases due to recall bias. When patients were asked about symptom onset, they were not asked specifically about onset of cough and fever. It is possible that patients reported the onset of a non-infectious cause of cough, rather than the onset of the accompanying fever or other symptoms. Additionally, although every effort was made to include all hospitalized infectious respiratory cases at our study sites, the inclusion criteria were biased toward certain symptomologies. We excluded patients <5 years who presented without cough or other respiratory symptoms, however, several studies have since demonstrated that children in this age group with influenza infection commonly present in the absence of respiratory symptoms [23, 24]. Data from the acute febrile illness arm of our surveillance site confirms this, as 10.4% of febrile children <5 years who presented without respiratory symptoms tested positive for influenza (unpublished data). Finally, the exact sensitivity, specificity, PPV, and NPV for the case definitions evaluated may differ across settings due to factors that would influence healthcare utilization rates and influenza incidence such as vaccination rates and prevalence of co-morbidities.

Data from this study support implementation of the 2014 WHO SARI case definition at local, national and global levels. In this study, the 2014 WHO SARI case definition captured the greatest number of ARI patients, influenza positive patients and ARI deaths as compared to the other case definitions examined. Additionally, this case definition is easy to implement and can be administered by trained healthcare workers rather than physicians. Consistent use of this case definition will establish national surveillance system baselines, a critical step for the rapid identification of unusual transmissibility, severity and/or impact features associated with emergent novel viruses through tools such as PISA. Additionally, the development and annual utilization of a national SARI sample testing scheme will allow for a targeted use of limited resources. Finally, a broader set of enrollment criteria for critically ill patients, such as those admitted to ICU, may increase capture of influenza and other respiratory infections with severe outcomes. Routine use of the 2014 WHO SARI case definition for hospital-based surveillance will provide a solid, globally comparable foundation on which to build needed response efforts for novel pandemic viruses.

## Supporting information

S1 File Infectious disease surveillance screening form. (PDF)

S2 File Infectious disease surveillance questionnaire. (PDF)

S1 Dataset (XLSX)
