# Supplementary material for: Comparison of common acute respiratory infection case definitions for identification of hospitalized influenza cases at a population-based surveillance site in Egypt
Source: PLoS One. 2021 Mar 25;16(3):e0248563. doi: 10.1371/journal.pone.0248563 (PMC7993808; doi:10.1371/journal.pone.0248563)
Supplement: S1 File — (PDF) [file pone.0248563.s001.pdf]

INFECTIOUS DISEASE SURVEILLANCE SCREENING FORM

بيان فحص حالات اشتباه الأمراض المعدية

SCREENING LABEL HERE<br>الرقم اللاصق للحالة المشتبهه	Today's Date تاريخ اليوم<br>Day اليوم — Month الشهر — Year السنة	Patient Admission ID Number:<br>رقم تذكرة الدخول	Surveillance Nurse Code:<br>كود ممرضة الترصد	Social Worker Code:<br>كود الأخصائية الاجتماعية

1. Patient admitted to hospital? ○ Yes (1) ○ ***No (0)*** هل تم حجز المريض بالمستشفى

Patient Name: اسم المريض الرباعى

2a. Damanhour Resident: ○ Yes (1) ○ ***No (0)*** مقيم دائما

2b. If no, what district? من أى مركز

3a. Date of Birth: DAY اليوم — MONTH الشهر — YEAR السنة تاريخ الميلاد

3b. ONLY if date of birth unknown, estimate age (years):
فى حالة عدم معرفة تاريخ الميلاد أذكر العمر لأقرب سنة

3c. If less than 2 years how many months:
للأطفال أقل من سنتين أذكر العمر بالشهور

4a. Admission Diagnosis: تشخيص الدخول

4b. Chief complaint or reason patient was selected for screening: سبب اختيار المريض كحالة اشتباه

****IF ANY RESPONSES ABOVE ARE UNDERLINED - THANK PATIENT FOR PARTICIPATING AND DISCONTINUE ALL SCREENING PROCEDURES**** لوكانت هناك اى اجابة سابقة من الإختيارات التى تحتها خط اشكر المريض ولا تستكمل البيان

5a. **Based on physician assessment, is there evidence of ACUTE INFECTION?** ○ Yes (1) ○ ***No (0)***

5b. **If NO, Explain and STOP ALL STUDY PROCEDURES:**

Surveillance Physician Code: كود طبيب الترصد

ACUTE RESPIRATORY INFECTION (ARI)

Is there evidence of RESPIRATORY INFECTION? ○ Yes (1) ○ No (0)

Patients ≥5 years ONLY

COMPLETE SECTION I ONLY

I.

6a. Signs of Acute Infection:

UNK	YES	NO	
	○	○	History of sudden onset of fever (with this illness)
	○	○	Current Fever ≥38°C
	○	○	Current Hypothermia <35.5°C
○	○	○	Abnormal WBC or differential

6b. Signs and Symptoms:

YES	NO	
○	○	Abnormal breath sounds
○	○	Tachypnea
○	○	Cough
○	○	Sputum production
○	○	Hemoptysis
○	○	Chest pain
○	○	Sore throat
○	○	Shortness of breath (dyspnea)

Children 1 month to <5years ONLY

MUST COMPLETE SECTIONS II, III AND IV FOR ALL CHILDREN 1 month to <5YRS

II.

7a. Signs of Acute Infection:

UNK	YES	NO	
	○	○	History of sudden onset of fever (with this illness)
	○	○	Current Fever ≥38°C
	○	○	Current Hypothermia <35.5°C
○	○	○	Abnormal WBC or differential

7b. Signs and Symptoms:

UNK	YES	NO	
	○	○	Abnormal breath sounds
	○	○	Tachypnea
	○	○	Cough
	○	○	Sputum production
	○	○	Hemoptysis
○	○	○	Chest pain
○	○	○	Sore throat
	○	○	Shortness of breath (dyspnea)

III.

8a. Does the patient have any of the following?

YES	NO	
○	○	Tachypnea
○	○	Cough
○	○	Difficulty Breathing

8b. Danger Signs:

UNK	YES	NO	
	○	○	Unable to drink or breastfeed
	○	○	Lethargic or unconscious
	○	○	Vomits everything
	○	○	Convulsions
	○	○	Nasal flaring
	○	○	Grunting
○	○	○	Oxygen saturation <90%
	○	○	Chest indrawing
	○	○	Stridor in a calm child

IV.

9a. Does the patient have:

YES	NO	
○	○	Tachypnea

9b. Does the patient have any of the following?

YES	NO	
○	○	Cough
○	○	Difficulty Breathing

If **'YES'** is selected AT LEAST ONCE in (a) **AND** **'YES'** is selected AT LEAST ONCE in (b) *in the same section*: **PATIENT ELIGIBLE** → ○ **Eligible**

ALL OTHER PATIENTS ARE NOT ELIGIBLE → ○ **NOT Eligible**

IEIP Code (Supervisor) **1st Data Entry Code** **2nd Data Entry Code**

Acute Infectious Neurological Disease (AIND)

Is there evidence of AIND? ◯ Yes (1) ◯ No (0)

Was there any evidence of AIND after the initial screening? ◯ Yes (1) → If Yes, Date of Onset: (At onset continue to screening section below): ◯ No (0)

DAY | MONTH | YEAR

ALL Patients ≥1 month

I.

12a. Signs of Acute Infection:

YES	NO	
◯	◯	Current Fever ≥ 38°C
◯	◯	History of fever during the course of this illness

12b. Signs of CNS Pathology in the past 14 days:

N/A UNK	YES	NO	
◯	◯	◯	Severe Headache
◯	◯	◯	Photophobia
	◯	◯	Stiff neck
	◯	◯	Kernig's Sign
	◯	◯	Brudzinski's Sign
◯	◯	◯	Bulging fontanel (<12 months)
◯	◯	◯	Poor Sucking
	◯	◯	Altered Consciousness
	◯	◯	Irritability
	◯	◯	Seizures
	◯	◯	Petechial or Purpural Rash
	◯	◯	Vomiting everything
	◯	◯	Toxic Appearance
	◯	◯	Lethargy
	◯	◯	Other Neurological Signs Specify:
	◯	◯	Altered mental status: Decreased/absent response to loud noise
	◯	◯	Altered mental status: Decreased/absent response to painful stimuli
	◯	◯	Altered mental status: Decreased/absent gaze fixation
	◯	◯	Altered mental status: Lack of recognition of familiar people/things
	◯	◯	Altered mental status: Seizure associated with loss of consciousness

If **'YES'** is selected AT LEAST ONCE in (a)
AND
'YES' is selected AT LEAST ONCE in (b)
in the same section:

PATIENT ELIGIBLE

ALL OTHER PATIENTS ARE NOT ELIGIBLE

◯ **Eligible**

◯ **NOT Eligible**

Is there evidence of AFI? ◯ Yes (1) ◯ No (0)

I

14a. Signs of Acute Infection:

YES	NO	
◯	◯	Current Fever ≥ 38°C
◯	◯	History of fever during the course of this illness

14b. Does the patient have any of the following?

YES	NO	
◯	◯	Eligible for acute respiratory infection
◯	◯	Eligible for acute infectious neurological disease
◯	◯	Evidence of septic arthritis
◯	◯	Evidence of otitis media
◯	◯	Evidence of urinary tract infection
◯	◯	Evidence of pyogenic soft tissue infection
◯	◯	Evidence of cellulitis
◯	◯	Other obvious source of infection

Specify: ______________________________

____

____

IF **'YES'** IS SELECTED AT LEAST ONCE in (b) the patient is **NOT ELIGIBLE**

If **'YES'** is selected AT LEAST ONCE in (a)
AND
ALL are **'NO'** in (b)

PATIENT ELIGIBLE

ALL OTHER PATIENTS ARE NOT ELIGIBLE

◯ **Eligible**

◯ **NOT Eligible**

Does patient have any of the following symptoms?

UNK	YES	NO	
◯	◯	◯	Myalgia
◯	◯	◯	Bruising
◯	◯	◯	Hemoptysis
◯	◯	◯	Erythema
◯	◯	◯	Rash If 'Yes', type of rash →
◯	◯	◯	Retroorbital Pain

◯ Hives ◯ Petechiae ◯ Purpura ◯ Macular
◯ Papular ◯ Other (specify): ______________

Acute Diarrheal Infection (ADI)

Is there evidence of ADI? ◯ Yes (1) ◯ No (0)

ALL Patients ≥1 month

COMPLETE SECTIONS I AND II

I.

10. Diarrhea:

YES	NO	
◯	◯	≥ 3 loose or liquid stools in a 24 hour period
◯	◯	≥ 1 loose or liquid stool AND dysentery

If **'YES'** is selected AT LEAST ONCE:

PATIENT ELIGIBLE

OR

II.

11a. Diarrhea:

YES	NO	
◯	◯	2 loose or liquid stools in a 24 hour period

11b. Does the patient have any of the following?:

YES	NO	
◯	◯	History of fever with this illness
◯	◯	Dysentery
◯	◯	Abdominal pain/cramps
◯	◯	Nausea
◯	◯	Vomiting

If **'YES'** is selected AT LEAST ONCE in (a)
AND
'YES' is selected AT LEAST ONCE in (b):

PATIENT ELIGIBLE

ALL OTHER PATIENTS ARE NOT ELIGIBLE

◯ **Eligible**

◯ **NOT Eligible**
