# Supplementary material for: Comparison of common acute respiratory infection case definitions for identification of hospitalized influenza cases at a population-based surveillance site in Egypt
Source: PLoS One. 2021 Mar 25;16(3):e0248563. doi: 10.1371/journal.pone.0248563 (PMC7993808; doi:10.1371/journal.pone.0248563)
Supplement: S2 File — (PDF) [file pone.0248563.s002.pdf]

INFECTIOUS DISEASE SURVEILLANCE QUESTIONNAIRE
استبيان لترصد الأمراض المعدية

STUDY LABEL HERE

ضع لاصق رقم الدراسة هنا

تاريخ استمارة الموافقة على الدراسة ☐☐-☐☐-☐☐☐☐ (اليوم / الشهر / السنة)

SCREENING LABEL HERE

الرقم اللاصق للحالة المشتبهه

كود الشخص الذي أخذ الموافقة ☐☐☐☐

كود طبيب الترصد ☐☐☐☐

موافقة المريض (أو الوصى)	لا (0)	نعم (1)	N/A (9)		لا (0)	نعم (1)	N/A (9)
على القيد بالدراسة (ARI) ؟	○	○	○	على استخدام العينات الحيوية فى المستقبل (ARI)؟	○	○	○
على القيد بالدراسة (AIND) ؟	○	○	○	على استخدام العينات الحيوية فى المستقبل (AIND)؟	○	○	○
على القيد بالدراسة (AFI) ؟	○	○	○	على استخدام العينات الحيوية فى المستقبل (AFI)؟	○	○	○
على القيد بالدراسة (ADI) ؟	○	○	○	على استخدام العينات الحيوية فى المستقبل (ADI)؟	○	○	○

IF PATIENT DID NOT CONSENT TO ENROLLMENT-STOP HERE إذا رفض المريض المشاركة فى الدراسة – لا تستكمل الاستبيان

Did patient transfer from a Damanhour hospital? هل تم تحويل المريض من مستشفى بدمنهور ○ Yes (1) ○ No (0) → **If 'YES' which hospital** (see attached codes) لو نعم اى مستشفى (أكتب كود المستشفى) ☐☐ → **If 'YES', was patient previously enrolled in this study for this episode?** هل تم ادراج المريض لهذه الحالة فى مستشفى اخرى ○ *Yes (1)* ○ No (0)

IF '*YES*' WAS ANSWERED FOR PREVIOUS ENROLLMENT- STOP HERE. في حالة الإجابة بنعم وتم ادراج المريض للدراسة في مستشفى آخرى يتم التوقف عن استكمال الدراسة

Attending Physician اسم الطبيب المعالج ☐

Date Questionnaire Administered تاريخ الأستبيان ☐☐-☐☐-☐☐☐☐ (DAY / MONTH / YEAR)

Interviewer (Code) كود القائم بالمقابلة ☐☐☐☐

Patient's Full Name اسم المريض الرباعى ☐

Village/Area code كود القرية / المنطقة ☐☐

Patient phone number رقم تليفون المريض ☐☐☐☐☐☐☐☐☐☐

Sex الجنس ○ Male (0) ذكر ○ Female (1) أنثى

Pregnancy Status هل يوجد حمل ○ Yes (1) ○ No (0) ○ N/A (9) ○ Unk (99) ○ Refuse (98)

Admission Date تاريخ الدخول ☐☐-☐☐-☐☐☐☐ (DAY / MONTH / YEAR)

Admission Department قسم الدخول ○ Pediatrics (1) أطفال ○ ICU (2) رعاية ○ Isolation (3)عزل ○ Male Ward (4)رجال ○ Female and Child Ward (5)نساء وأطفال

Adjusted Temperature درجة الحرارة ☐☐.☐ °C ○ Admission (1) وقت الدخول ○ At Enrollment (2) وقت الفحص

Respiratory Rate (per minute) معدل التنفس (فى الدقيقة) ☐☐☐ ○ Admission (1) وقت الدخول ○ At Enrollment (2) وقت الفحص

Oxygen Saturation (At Enrollment) تشبع الأكسجين ☐☐☐ %

Before today, number of days with symptoms عدد أيام ظهور الأعراض بإستثناء اليوم ☐☐

Was patient hospitalized in the last 6 days? هل تم حجز المريض فى الأيام الستة الماضية ○ Yes (1) ○ No (0) ○ Unk (99) ○ Refuse (98)

→ If yes, number of days hospitalized: عدد أيام الحجز ☐☐

If in Damanhour - Hospital CODE: كود المستشفى فى دمنهور ☐☐

If OTHER- Hospital Name: اسم المستشفى خارج دمنهور ☐

Ever travelled outside of Egypt? هل سافرت للخارج ○ Yes (1) ○ No (0) ○ N/A (9) ○ Unk (99) ○ Refuse (98)

→ If 'Yes', where? اين ☐ **Date:** ☐☐-☐☐☐☐ (MONTH / YEAR)

Where اين ☐ **Date:** ☐☐-☐☐☐☐ (MONTH / YEAR)

Where? اين ☐ **Date:** ☐☐-☐☐☐☐ (MONTH / YEAR)

Vaccination Status: موقف التطعيم

Seasonal Influenza تطعيم الأنفلونزا الموسمى	○ Yes (1)	○ No (0)	○ Unk (99)	○ Refuse (98)
H1N1/09 تطعيم انفلونزا الخنازير	○ Yes (1)	○ No (0)	○ Unk (99)	○ Refuse (98)
HIB تطعيم الهيموفيلس	○ Yes (1)	○ No (0)	○ Unk (99)	○ Refuse (98)
Strep pneumo تطعيم ستربت نيموكوك	○ Yes (1)	○ No (0)	○ Unk (99)	○ Refuse (98)
Meningococcal تطعيم الحمى الشوكية الوبائية	○ Yes (1)	○ No (0)	○ Unk (99)	○ Refuse (98)
Yellow Fever تطعيم الحمى الصفراء	○ Yes (1)	○ No (0)	○ Unk (99)	○ Refuse (98)

ANTIBIOTIC USE
المضاد الحيوى

Patient took antibiotics 3 days before هل تم أخذ المضاد فى ال 3 أيام السابقة للدخول :

○ Yes (1) ○ No (0) ○ Unk (99) ○ Refuse (98)

If 'Yes'- Code: **كود** ☐☐☐ ○ Verbal (1) شفهيا ○ Box (2) رؤية ☐☐☐ ○ Verbal (1) شفهيا ○ Box (2) رؤية

If yes, but no code, specify: لو نعم ولا يوجد كود أذكره ☐

Patient given antibiotics at the hospital BEFORE specimen collection: هل تم أعطاء المضاد فى المستشفى قبل أخذ العينات

○ Yes (1) ○ No (0) ○ Unk (99)

If 'Yes'- Code: كود المضاد ☐☐☐ ☐☐☐ ☐☐☐

If yes, but no code, specify: لو نعم ولا يوجد كود أذكره ☐

Pre-existing chronic diseases: هل يوجد امراض مزمنة حاليا ○ Yes (1) ○ No (0) ○ Unk (99) ○ Refuse (98)

If 'Yes', specify ☐ أذكر المرض

If 'Yes', specify ☐ أذكر المرض

If 'Yes', specify ☐ أذكر المرض

IEIP Code (Supervisor) ☐☐☐☐

1st Data Entry Code ☐☐☐☐

2nd Data Entry Code ☐☐☐☐

OUTCOME-ALL PATIENTS

Did the physician prescribe antibiotics during hospitalization? (AFTER specimen collection) ◯ Yes (1) ◯ No (0)

If 'Yes'- Codes: [ ][ ][ ] [ ][ ][ ] [ ][ ][ ] / [ ][ ][ ] [ ][ ][ ] [ ][ ][ ]

If yes, but no code, *specify*: [ ]

Total White Blood Cell Count ◯ Not done (0) [ ][ ][ ][ ][ ][ ]

White Blood Cell Differential ◯ Not done (0) ◯ Done (1)

If done:

- **a. Neutrophils** [ ][ ] . [ ] %
- **b. Lymphocytes** [ ][ ] . [ ] %
- **c. Monocytes** [ ][ ] . [ ] %
- **d. Band** [ ][ ] . [ ] %

Outcome ◯ Discharge (1) ◯ Abscond (2) ◯ Transfer (3) ◯ Death (4) ◯ Unk (99)

If 'Transfer', to which hospital?

In Damanhour - Hospital CODE: [ ][ ]

OTHER- Hospital Name: [ ]

Outcome Date [ ][ ]-[ ][ ]-[ ][ ][ ][ ]
DAY MONTH YEAR

Discharge Diagnosis ◯ Unk (99)

[ ]
[ ]
[ ]

Was patient in ICU for this episode? ◯ Yes (1) ◯ No (0) → **If yes, how many days?** [ ][ ]

Was patient ventilated for this episode? ◯ Yes (1) ◯ No (0) → **If yes, how many days?** [ ][ ]

ACUTE RESPIRATORY ILLNESS (ARI)

Oxygen saturation ◯ Not done (0) [ ][ ][ ] %

TB Smear ◯ Not done (0) ◯ Done (1)
If done, RESULTS: ◯ Negative (0) ◯ Positive (1)

Pleural Tap Culture ◯ Not done (0) ◯ Done (1)
If done, RESULTS: ◯ Negative (0) ◯ Positive (1)
If Positive, *Specify*: [ ]

Chest X-Ray ◯ Not done (0) ◯ Done (1)
If done, RESULTS: *(check all that apply)*
◯ Normal ◯ Pneumatocele ≤1
◯ Infiltrate ◯ Effusion
◯ Consolidation ◯ Unknown
◯ Other *Specify:* ◯ Cavitation
[ ]

ACUTE INFECTIOUS NEUROLOGICAL DISEASE (AIND)

Lumbar Puncture ◯ No CSF obtained (0) ◯ CSF obtained (1)
If obtained:
Appearance: ◯ Clear (1) ◯ Turbid (3) ◯ Hazy (2) ◯ Bloody (4)
Total Leukocytes [ ][ ][ ][ ][ ][ ]
Lymphocytes [ ][ ][ ] . [ ] %
Neutrophils [ ][ ][ ] . [ ] %
Protein [ ][ ][ ] gm/dl
Glucose [ ][ ][ ] mg/dl
Erythrocytes [ ][ ][ ][ ][ ] mm^3

Gram Stain ◯ Negative (1) ◯ Positive (2) ◯ Not done (0)
If Positive: ◯ Bacilus Gram Negative (1) ◯ Cocci Gram Negative (3)
◯ Bacilus Gram Positive (2) ◯ Cocci Gram Positive(4)
◯ Other (5): [ ]

CSF Culture: ◯ Negative (1) ◯ Positive (2) ◯ Not done (0)
If Positive, *Specify:* [ ]

Serum Glucose ◯ Not done (0) [ ][ ][ ][ ] mg/dl

CT Scan ◯ Not done (0) ◯ Normal (1) ◯ Abnormal (2)

MRI ◯ Not done (0) ◯ Normal (1) ◯ Abnormal (2)

Does the physician suspect TB? ◯ No (0) ◯ Yes (1) ◯ Unknown (99)

ACUTE DIARRHEAL INFECTION (ADI)

Stool Sample ◯ Not Collected (0) ◯ Collected (1)

If collected, Macro-analysis:
- Blood ◯ Negative (0) ◯ Positive (1)
- Mucus ◯ Negative (0) ◯ Positive (1)

Micro-analysis:
- G. lamblia ◯ Negative (0) ◯ Positive (1)
- E.histolytica ◯ Negative (0) ◯ Positive (1)
- Cryptosporidium ◯ Negative (0) ◯ Positive (1)
- Other ◯ Negative (0) ◯ Positive (1)

Specify: [ ]

Specify: [ ]

ACUTE FEBRILE ILLNESS (AFI)

Blood Culture ◯ Not done (0) ◯ Done (1)
If done, RESULTS: ◯ Negative (0)
If Positive, Specify Result: [ ]

Malaria Smear ◯ Not done (0) ◯ Done (1)
If done, RESULTS: THIN smear: ◯ Negative (0) ◯ Positive (1)
THICK smear: ◯ Negative (0) ◯ Positive (1)

Urine Culture ◯ Not done (0) ◯ Done (1)
If done, RESULTS: ◯ Negative (0) ◯ Positive (1)
If Positive, Specify Result: [ ]

Urinalysis ◯ Not done (0) ◯ Done (1)
If done, RESULTS: ◯ Normal ◯ Pyuria [ ][ ][ ] /HPF
◯ Hematuria [ ][ ][ ] /HPF

ESR ◯ Not done (0) ◯ Done (1)
If done, RESULTS: [ ][ ][ ] 1ST Hour

If done, RESULTS:
C-Reactive Protein ◯ Not done (0) ◯ Done (1)
[ ][ ][ ] mg/l
